# MicroRNAs in Lupus Nephritis–Role in Disease Pathogenesis and Clinical Applications

**DOI:** 10.3390/ijms221910737

**Published:** 2021-10-04

**Authors:** Benjamin Y. F. So, Desmond Y. H. Yap, Tak Mao Chan

**Affiliations:** Division of Nephrology, Department of Medicine, Queen Mary Hospital, The University of Hong Kong, Hong Kong; soyufaibenjamin@gmail.com (B.Y.F.S.); dtmchan@hku.hk (T.M.C.)

**Keywords:** microRNA, lupus nephritis, epigenetics, pathogenesis, clinical applications

## Abstract

MicroRNAs (miRs) are non-coding small RNAs that act as epigenetic modulators to regulate the protein levels of target mRNAs without modifying the genetic sequences. The role of miRs in the pathogenesis of lupus nephritis (LN) is increasingly recognized and highly complex. Altered levels of different miRs are observed in the blood, urine and kidney tissues of murine LN models and LN patients. Accumulating evidence suggests that these miRs can modulate immune cells and various key inflammatory pathways, and their perturbations contribute to the aberrant immune response in LN. The dysregulation of miRs in different resident renal cells and urinary exosomes can also lead to abnormal renal cell proliferation, inflammation and kidney fibrosis in LN. While miRs may hold promise in various clinical applications in LN patients, there are still many potential limitations and safety concerns for their use. Further studies are worthwhile to examine the clinical utility of miRs in the diagnosis, disease activity monitoring, prognostication and treatment of LN.

## 1. Introduction

Systemic lupus erythematosus (SLE) is an autoimmune disease characterized by loss of tolerance to various self-antigens, leading to immune-mediated tissue injury. Lupus nephritis (LN) is one of the most devastating manifestations of SLE, affecting up to 70% of patients with SLE [1,2]. Immune complexes are deposited in the mesangial, subepithelial and subendothelial regions of the renal glomeruli of patients with LN, and kidney injury occurs due to activation of various inflammatory, proliferative and fibrotic pathways. The different patterns of immune complex accumulation define the different histopathological subtypes of LN and their associated clinical manifestations [3]. Patients may present with variable degrees of proteinuria, microscopic haematuria, hypertension and loss of kidney function. Aggressive immunomodulatory treatment is often indicated to alleviate debilitating symptoms and prevent complications including development of end-stage kidney disease (ESKD) and death, particularly in active, proliferative forms of LN. Unfortunately, despite multimodal therapy with corticosteroids, other immunosuppressants and biologic agents, a significant proportion of patients remain refractory to standard treatments. Many patients suffer from treatment-related adverse events including opportunistic infections, infertility and development of secondary malignancies. LN thus remains an important cause of ESKD and its related morbidities, often among young, fertile and productive individuals [1,2]. 

Both genetic and environmental factors implicated in the pathogenesis of LN have been extensively investigated. In the past decade, the role of epigenetics—heritable changes in gene function that occur without a change in genetic code—in the pathogenesis of SLE has become increasingly recognized. Various mechanisms of epigenetic regulation of gene expression, including DNA methylation/acetylation, histone and non-histone protein modifications, and non-coding RNAs that regulate messenger RNA (mRNA) expression at the post-transcriptional level, have been studied in the context of SLE and associated with specific phenotypes of SLE [4,5,6,7,8].

Non-coding RNAs are arbitrarily divided into long non-coding RNAs (lncRNAs) and microRNAs (miRs) depending on their length. miRs function by binding to the 3′ untranslated region (UTR) of mRNA leading to translation repression and/or mRNA degradation, typically resulting in decreased production of specific proteins [9,10,11]. The biogenesis of miRs is a multi-stage process. First, the primary transcript, known as primary microRNA (pre-miRNA) is formed in the nucleus by RNA polymerase II and III. Maturation of pri-miRNA is mediated by the RNA-binding protein complex DGCR8 (DiGeorge syndrome critical region 8) and Drosha ribonuclease, a nuclear RNase III enzyme, forming a small hairpin precursor microRNA (pre-miRNA). The pre-miRNA is then exported into the cytoplasm via Exportin 5 (EXP5) and its cofactor Ran-GTP. Once in the cytoplasm, the enzyme Dicer cleaves the pre-miRNA to produce a small double-strand RNA (dsRNA) composed of a guide strand and a passenger strand. The passenger strand is degraded and the guide stranded, which is then loaded onto the RNA_induced silencing complex (RISC) containing Argonaut (Ago) protein. The miR-RISC complex, once formed, is capable of silencing mRNA targets at the pre-translational, co-translational and post-translational levels [12].

Importantly, miRs are detectable in both human cell lines and body fluids, including the blood, urine and cerebrospinal fluid, and miR signatures in tissues and in body fluids that are specific for particular diseases have been defined [13]. Profiling of differentially expressed miRs can be performed using quantitative polymerase chain reaction (qPCR) methods, in situ hybridization (ISH) or RNA sequencing by next-generation sequencing (NGS) on various specimens [14,15,16]. These studies lend insight into the epigenetic factors contributing to the pathogenesis of various diseases, and may even translate into practical applications in diagnosis and treatment. 

A growing body of evidence from both animal and human studies suggests that expression of miRs is altered in SLE [5,6,7,8]. Whereas previous reviews have covered the epigenetic landscape in SLE in general, this review will focus specifically on end-organ involvement in the kidney, elaborating on the epigenetic regulatory mechanisms salient to and specific for the development and evolution of LN. The clinical applications of these important miRs in individualized diagnosis, disease activity monitoring and treatment of LN patients will also be discussed. 

## 2. MiRs in the Circulation and Peripheral Blood Mononuclear Cells (PBMCs)

In this case, miRs are found in the circulation, including in cells but also in extracellular vesicles, or as part of miR-protein complexes. A large number of miRs are differentially expressed in the plasma as well as in PBMCs in patients with SLE; and some studies have correlated circulating miR levels as well as miR expression in PBMCs with various clinical outcomes or biomarkers in LN [17,18,19]. The roles of many of these miRs are not fully known, exemplifying the complexity of epigenetic regulation in LN. 

Various inflammatory pathways contribute to the end-organ manifestations of SLE, including LN. The NF-κB and type I interferon (IFN) pathways are important inflammatory pathway in SLE and LN. These inflammatory cascades can be modified by miRs through regulation of the innate and adaptive immune systems. For example, miR-146a is typically induced by toll-like receptor (TLR) activation via lipopolysaccharide (LPS) stimulation and plays a crucial role in regulation of the NF-κB and type I interferon (IFN) pathways by targeting signal transducers such as TNF receptor-associated factor 6 (TRAF6), IL-1 receptor-associated kinase 1 (IRAK1), IFN regulatory factor 5 (IFN-5) and signal transducer and activator of transcription-1 (STAT1). In LN patients, miR-146a expression in PBMCs shows an inverse relationship with inflammatory markers that are closely associated with the NF-κB pathway (e.g., IL-1β, IL-6, IL-8 and TNF-α), suggesting that downregulation of circulating miR-146a promotes immune activation and inflammation in LN [20]. Profiling of circulating miR expression in SLE patients with and without LN demonstrated a negative correlation between miR-146a expression and clinical disease activity and interferon scores [21]. In agreement with these results, administration of a miR-146a mimic to LN mice impaired both the classical and non-classical NF-κB signaling pathways, and was associated with reduced renal inflammation [22]. Interestingly, among patients with confirmed LN, blood levels of miR-146a was higher in some studies [23,24], but lower than controls in others [20,25,26,27], although none of these studies had sufficiently large sample sizes to draw definitive conclusions. Other studies have shown that miR-124 is similarly downregulated in patients with active LN, resulting in increased expression of its mRNA target, TRAF6. The serum level of miR-124 was thus negatively correlated with IL-1β, IL-6 and TNF-α [28]. The circulating levels of miR-199a-1 and miR-125b-5p, which are both negatively regulated by NF-κB, were elevated in patients with class IV LN in one study [19]. The plethora of studies illustrate that multiple miRs may either mediate activation of, or be regulated by, the IFN/NF-κB inflammatory pathways, and that altered expression of these miRs may contribute to inflammation in LN. 

Macrophage migration inhibitory factor (MIF) is an upstream regulator of innate immunity in human macrophages, promoting expression of cytokines such as IL-1β, IL-6, IL-8 and TNF-α by suppressing activation-induced apoptosis, and may counter-regulate the effects of corticosteroids. In SLE patients, MIF expression is increased and correlated with reduced serum levels of miR-654, which is a regulator of not only MIF but also its receptor CD44. miR-654 can dampen the effects of MIF-mediated expression by decreasing the tyrosine phosphorylation of mitogen-associated protein kinases (MAPK) and Akt. Reduced miR-654 levels were additionally correlated with greater 24-h urine protein and high serum creatinine [29]. Adding to that, miR-451a, which also targets MIF expression, was downregulated in serum exosomes in patients with SLE, particularly in those with LN and higher SLE disease activity index (SLEDAI) scores [30]. Given the role of serum exosomes in intercellular communication, it is possible that MIF mediates crosstalk between the innate and adaptive immune systems.

Altered expression of miRs in T and B cells lead to both quantitative and qualitative defects in various lymphocyte subsets, resulting in activation of inflammatory pathways and end-organ damage. Expression of miR-21 in PBMCs in patients with LN was greater than both SLE patients without LN and healthy controls and was higher in active compared with inactive disease [31,32]. Expression of miR-21 was associated with higher SLEDAI scores, but there was no consistent relationship with serological markers of SLE disease activity including levels of complement C3 or C4 [31]. miR-21 was associated with activation of pro-inflammatory protein programmed cell death 4 (PDCD4)/IL-10, phosphatase and tensin (PTEN) homolog/forkhead box O (FoxO) and SMAD/transforming growth factor-β (TGF-β) pathways. In addition to the aforementioned mechanisms, miR-21 as well as miR-148a were found to interact with other epigenetic mechanisms by downregulating DNA methyltransferase I (DNMT1) expression in T cells, contributing to DNA hypomethylation and consequent T cell dysfunction [33]. Overexpressed miR-21 tended to polarize T cell differentiation towards the T_H_17 phenotype at the expense of regulatory T cells, and also regulates T-cell co-stimulation including CD40:CD154 and CD28:CD80/86. One study showed decreased miR-10a-3p in PBMCs of LN patients, which increased the T_H_17/Treg ratio in CD4^+^ T cells via upregulation of regenerating islet-derived 3 α (REG3A), and also activated the JAK2/STAT3 signalling pathway downstream [34]. In murine lupus models, systemic administration of miR-10a-3p reversed these changes and also improved renal function, 24-h urine protein, markers of renal injury such as kidney injury molecule-1 (KIM-1) and decreased anti-dsDNA levels. In T cells of SLE patients, decreased miR-145 upregulated expression of STAT1 while increased miR-224 decreased expression of apoptosis inhibitory protein 5 (API5), enhancing T cell apoptosis after stimulation. In this study, the elevated expression level of STAT1 was significantly associated with LN, even after adjustment for SLEDAI scores [35]. miR-371b-5p was significantly increased in both CD4^+^ and CD8^+^ T cells while miR-5100 expression was increased in CD4^+^ T cells in SLE patients compared to healthy controls and patients with rheumatoid arthritis (RA) [36]. The increased expression of these miRs were significantly more pronounced in active SLE, particularly in those patients with kidney involvement. Taken together, these findings suggest that these two miRs mediate their pathogenic effects in LN via aberrant function of CD4^+^ and CD8^+^ T cells. Finally, in murine models, miR-223 deficiency induced expression of CD4^+^ T cells expressing sphingosine-1-phosphate receptor (S1pr1), which tend to migrate to the spleen and stimulate macrophage infiltration into the kidney tissue [37].

In addition to modulating T cell differentiation, miR-155 is also crucial for B cell maturation and development and is correlated with peripheral B cell frequency as well as extrafollicular formation in secondary lymphoid organs. miR-155 was decreased in LN patients in tandem with a lowered glomerular filtration rate (GFR) [27], echoing other studies involving SLE patients with and without LN [17,38], although these results were not consistently reproduced in other studies involving LN patients [23,31]. Used in conjunction with other miRs, plasma circulating levels of miR-155 was used to discriminate LN patients from healthy controls in a small study [23]. The B cell signature can also be modified by miR-29c, which was reduced in B cells of patients with LN, through its predicted gene targets including tumor necrosis factor (TNF), IL-6 and B-cell activating factor (BAFF) [39]. On the contrary, miR-145 expression is upregulated in B cells of LN patients, possibly contributing to reduced B cell proliferation and B cell lymphopoenia [39]. As part of a composite score in conjunction with other miRs and anti-dsDNA levels, miR-145 was highly predictive of active LN in paediatric patients [40]. In patients with SLE, TLR7 activation decreased miR-15b in B cells, thereby increasing cyclin D3, an important mediator of B cell proliferation, development and differentiation [41]. Correspondingly, decreased plasma levels of miR-15b in LN patients were associated with increased disease activity and lower GFR [38]. Furthermore, higher miR-148a levels in serum and in B cells of LN patients have been shown to lower BTB and CNC homology 1 (BACH1), BTB and CNC homology 2 (BACH2) and paired box 5 (PAX5) expression in B cells, resulting in a lower percentage of circulating naïve B cells and a higher memory B cell-to-naïve B cell ratio, and is associated with development of multiple relapses in patients with LN [42]. Even though other miRs, such as miR-22, miR-18b, miR-345 and miR-365, are differentially overexpressed in B cells of patients with LN compared to controls and presumably should modify B cell function or expression, their exact biological and pathogenic role in LN remains to be elucidated [38,39]. 

In recent years, other lymphoid cells with regulatory capabilities including B regulatory (Breg) cells and natural killer (NK) cells have been found to play a key role in the pathogenesis of LN. For example, NK cell cytotoxicity against self-antigens is inhibited in patients with SLE, due to development of antibodies against the killer Ig-receptor (KIR), and this is associated with more severe end-organ manifestations including LN [43]. In vitro studies show that miR-146a-5p may be involved in the regulation of killer Ig-receptor (KIR) expression [44], and perturbations of miR-150 may affect the development of NK and invariant NKT (iNKT) cells [45]. The contribution of these miRs to regulatory cell dysfunction in the context of LN requires clarification in future studies.

Beyond effects on the innate and adaptive immune systems, many miRs mediate their effects at the tissue level, but are upregulated in the serum and hold promise as non-invasive diagnostic or prognostic biomarkers. For example, serum miR-130-3p levels were increased in early stage LN regardless of SLEDAI scores and was associated with renal damage, 24-h urine protein excretion and chronicity index (CI) on kidney biopsy samples [46]. miR-423 is associated with epithelial-mesenchymal transition (EMT) and renal fibrosis and elevated levels of miR-423-5p was demonstrated in PBMCs of LN patients across different races [18,32]. Similarly, miR-182-5p was particularly upregulated in LN patients with high CI, and was associated with lowered serum levels of FoxO1, a protective factor against tubulointerstitial fibrosis and tubular atrophy in different kidney diseases [47]. The mechanisms underlying the effects on renal damage and fibrosis are further explored in sections below. 

**Figure 1:**
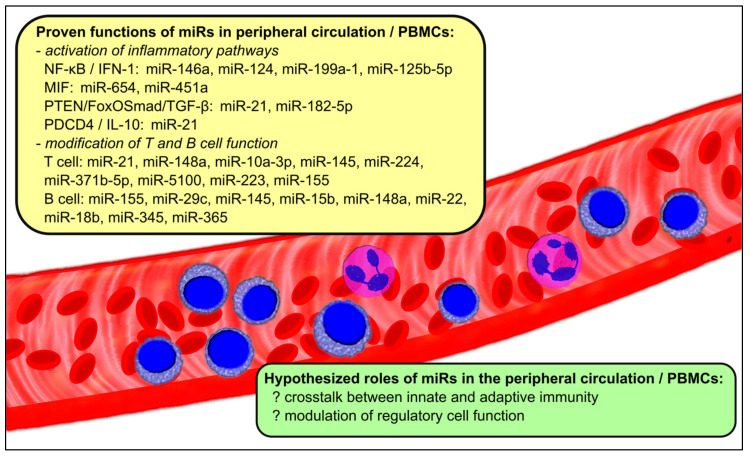
The roles of miRs in the peripheral circulation and PBMCs in LN.

Some of the key signal transduction pathways involved in the pathogenesis of LN, and the miR correlates, are summarized in Table 1 and illustrated in Figure 1. Yet the list of miRs described above is far from exhaustive. Indeed, over a hundred different miRs have been identified as being differentially expressed in the circulation or in PBMCs of patients with LN [17,18,19,25,48]. For many of these candidate miRs, biological targets may be predicted from bioinformatics databases, but these will no doubt have to be further verified with in vitro and in vivo studies [49]. Confounding the picture, however, the profile of miR expression in the circulation of LN patients is heterogeneous and at times conflicting when comparing across different studies, possibly due to heterogeneity in the racial and ethnic composition of LN populations studied, different states of disease activity, the effects of immunosuppressive treatments, and other technical difficulties and variations. 

Significant heterogeneity may be introduced by the technical challenges of miR extraction from human plasma or serum. First, high levels of endogenous RNases are present, such that rapid RNA degradation occurs if RNA extraction methods cannot rapidly inactivate RNase [16,53]. Relevant to the context of LN, circulating miR is known to decrease in advanced chronic kidney disease (CKD) due to high circulating levels of RNases, making comparison and extrapolation of study results difficult across the different stages of CKD among patients with LN and in animal models [54]. Second, variation may occur due to technical issues during specimen processing including centrifugation conditions, white cell counts and red cell haemolysis [52,55,56]. Third, whereas many studies perform total RNA extraction from whole blood on the assumption that most detected miR are derived from PBMCs, studies have shown that miR levels quantified from isolated PBMCs varies from the whole blood, due to the presence of other miR-containing cells, such as erythrocytes [57]. The cell-free fraction of miRs, in serum exosomes/vesicles or with protein complexes, also needs to be accounted for. Studies also differ as to whether sera or plasma is used for analysis. Inconsistencies in extraction protocols may account for the discrepant results observed in the studies in LN. Finally, it remains difficult to scale up to high-throughput sequencing for highly proteinaceous liquid specimens such as human plasma or serum [16]. These technicalities notwithstanding, the many studies cited and described above suggest that circulating miRs remain attractive candidates as disease biomarkers for diagnosis, prognosis and monitoring in LN. Even though one single miR may not be sufficiently diagnostic on its own, a panel of pathogenic miRs may show good performance in distinguishing between different phenotypes, activity and stages of LN. Clearly, translation to clinical use would require the design of robust studies, with standardization of pre-analytic and analytic variables, to validate specific miR signatures to guide decisions of clinical management.

## 3. MiRs in the Kidney Tissue

The miR profile in kidney biopsy samples from LN patients was first reported in 2009, though this was a small study with only 5 patients with inactive, class II LN [58]. Since then, a plethora of studies have shown that many miRs are differentially expressed in various renal cells in both human subjects and murine models, with only limited overlap with the miR signature in the systemic circulation [49,51,59,60]. LN is a heterogeneous disease involving immune-mediated injury to different cell types in the kidney, including mesangial cells and podocytes in the glomeruli, tubulointerstitial cells as well as vascular smooth muscle and endothelial cells, among others. Accumulating evidence also demonstrated that these resident renal cells also have key pathogenic roles in the inflammatory and fibrotic process of LN [61,62,63,64,65,66,67,68]. miRs are intricately connected with inflammation, dysfunction, remodeling and fibrosis involving different kidney cell types; some of those shown to be correlated with activation of various signal transduction pathways are presented in Table 2.

Even though the pattern of expression of miRs in kidney tissues is not identical to that in the peripheral blood, the signal transduction pathways involved are demonstrably similar. In a human cell line model of LN, miR-199a, which is a regulator of IκB kinase-β (IKKβ) expression, was elevated and associated with NF-κB pathway activation with enhanced TNF-α and IL-1β secretion. The NF-κB associated inflammation was negatively regulated by Klotho, a membrane protein constitutively expressed in the human kidney possessing antioxidant and renoprotective properties and which was in turn downregulated by miR-199a in vitro [69]. Let-7 miRs, including let-7a and let-7e, were also overexpressed in kidney tissue samples from patients with LN, and let-7 overexpression was associated with enhanced NF-κB activity in human embryonic kidney cells. These effects were likely mediated by repression of the expression of TNF alpha induced protein 3 (TNFAIP3), an ubiquitin-editing enzyme that functions as a terminator of NF-κB activation following stimulation by exogenous pathogens or proinflammatory cytokines, by let-7 upregulation [70]. Other miRs are also involved in this same pathway: TNFAIP3 is bound to TNFAIP3 interacting protein (TNIP2), which is in turn targeted by miR-663a/miR-423-5p. In this case, miR-663a/miR-423-5p were overexpressed in kidney biopsies from LN patients, and was associated with reduced TNIP2 expression, and increased IL-1β, IL-6 and TNF-α secretion [72]. Let-7a may also function in post-transcriptional regulation of IL-6 by increasing the expression of tristetraprolin (TTP), an RNA-binding protein with 5 potential binding regions in the 3′ UTR of IL-6, as shown in NZB/W lupus mice with active disease [71]. 

Meanwhile, miR-130b expression is reduced in kidney biopsy specimens from LN patients, in contrast to its expression in the serum. In both human subjects and in murine models, miR-130b was shown to negatively regulate activation of the type I IFN pathway by inhibiting STAT1 tyrosine phosphorylation, reducing the expression of several IFN-inducible genes at both mRNA and protein levels. Specifically, IFN regulatory factor 1 (IRF-1) was identified as a direct target of miR-130b in mesangial cells in the kidney; silencing of IRF-1 with an siRNA effectively impaired type I IFN signaling in mesangial cells [73]. IRF-1 induced expression of human epidermal growth factor receptor 2 (HER-2), which downregulated miR-26a and miR-30b, thereby reversing the repression of genes involved in the cell cycle and promoting cell proliferation. Mesangial cells exposed to trastuzumab, a humanized monoclonal antibody targeted against HER-2, had restored levels of miR-26a and miR-30b and reduced mitosis and cell proliferation, validating the importance of this pathway [75]. Upstream, exogenous administration of miR-130b may hold therapeutic potential as treatment of LN mice with a miR-130b agomir reduced proteinuria and attenuated mesangial proliferation, though there was no effect on immune complex or complement deposition, suggesting a direct modulation of mesangial cell response to immune-mediated injury [73].

In addition to its effect on the IFN pathway, dysregulation of miR-130b may also contribute to mesangial cell proliferation via effects on PTEN, a phosphatase protein involved in cell cycle regulation that is a direct target of miR-130b. In contradiction with other studies, however, a small study showed that miR-130b in kidney tissues was increased, rather than decreased in patients with LN and was negatively correlated with PTEN [74]. It is unclear whether these discordant results may be due to different patient populations studied or differences in methods. MiR-198 can also bind directly to the PTEN 3′ UTR. Increased expression of miR-198 was observed in the kidney biopsy specimens of LN patients and was also correlated with disease activity, inhibition of PTEN, and enhanced mesangial cell proliferation [76]. These findings were corroborated in other studies that reported increased miR-198 expression in both the glomeruli and tubulointerstitium of LN patients [51].

Other miRs and proteins are involved in the pathogenesis of mesangial cell proliferation, a key histologic feature of active LN. For example, miR-133 was significantly decreased in kidney tissue in LN, with increased levels of Lim and SH3 protein 1 (LASP1), a structural scaffolding protein and adhesion adaptor protein originally detected in solid tumors, in human mesangial cells. This was associated with cell cycle abnormalities, with decreased cells in G1 phase and increased cells in S phase, increasing the rate of cellular proliferation [82]. A possible role is suggested for miR-16, as administration of miR-16 agomirs to LN mice inhibited mesangial cell proliferation by binding of miR-16 to differentially expressed in chondrocytes 2 (DEC2), inactivating intrarenal TLR4 signalling pathways [83]. In a separate study, miR-371-5p was downregulated in kidney tissues of LN patients. Hypoxia-inducible factor-1 α (HIF-1α), which can promote mesangial cell growth in LN, was identified as a direct target of miR-371-5p; as a corollary, restoration of miR-371-5p levels suppressed mesangial cell proliferation in vitro [84]. 

Multiple other signal transduction pathways may be involved in the pathogenesis of intrarenal inflammation in LN. In one study, miR-146a was increased in the glomeruli and correlated with gene expression of fibroblast growth factor-inducible 14 (Fn14), a key component of the TNF-like weak inducer of apopotosis (TWEAK)/Fn14 pathway, which may activate various proinflammatory intracellular signaling pathways and induce glomerular and tubular infiltration of macrophages [51]. The kallikrein-kinin system may also mediate renal inflammation in various kidney diseases. Among an array of differentially expressed miR in kidney biopsy samples from LN patients, miR-422a was most upregulated in active LN, and was found to directly downregulate kallikrein-related peptidase 4 (KLK4) mRNA. KLK4, which is proposed to have renoprotective properties, was reduced in renal mesangial and tubular epithelial cells in both human LN tissue and in murine LN models [59].

Vascular smooth muscle cell (VSMC) differentiation and proliferation in the kidney are modified by the miR-143/145 cluster of miRs. miR-145 was expressed in epithelial cells of proximal convoluted tubules and VSMCs of renal vessels in children with LN, and its expression decreased with increasing vascular damage. miR-145 protected against vascular damage in LN by inhibiting proliferation, migration, differentiation and phenotypic transformation of human VSMCs induced by platelet derived growth factor (PDGF)-BB [87]. VSMCs showed different phenotypes depending on miR-145 expression levels, with a physiological contractile phenotype linked with high expression levels and a proliferative phenotype at lower expression level. This was associated with pathological features of vascular damage such as thickened tunica media vasorum and reflected by non-invasive biomarkers including serum and urinary vascular endothelial growth factor (VEGF) levels. These features were most prominent in patients with severe class IV or class IV + V LN [85], which correlates with previous data showing that circulating VEGF levels is associated with SLE, particularly active LN [88,89]. VEGF expression is correlated with accelerated atherosclerosis in SLE [90], but whether miR-145 directly contributes to its pathogenesis is not yet known.

Chronic inflammation in the kidney is inevitably followed by fibrosis, with attrition of nephrons and progressive loss of kidney function. Transforming growth factor β1 (TGF-β1) and its associated pathways are important regulators of tissue fibrosis, including in the kidney. miR-150 is upregulated in the kidney tissue in LN patients, particularly in proximal renal tubular cells but also in mesangial cells and podocytes, and promotes renal fibrosis by downregulating suppressor of cytokine signal 1 (SOCS1) and increasing expression of profibrotic factors including fibronectin, COL1, COL3 and TGF-β1 [77,78]. Studies have demonstrated a positive feedback loop between TGF-β1 and miR-150. Intrarenal levels of miR-150 are directly correlated with CI in kidney samples from LN patients, and may predict subsequent development of ESKD. Extrapolating from these studies, administration of locked nucleic acid (LNA)-anti-miR-150 to LN mice suppressed renal miR-150 levels and reversed the profibrotic milieu, with reduced TGF-β1, α-smooth muscle antibody, and fibronectin and increased SOCS1. These changes were also accompanied by decreased renal proinflammatory cytokines such as IL-6 and TNF-α [77]. In a murine model of LN, miR-410 was underexpressed in kidney tissue, and was associated with both increased IL-6 and TGF-β1; administration of a miR-410 inhibitor suppressed fibrosis by reversing these effects [79]. Likewise, downregulation of miR-183 in the kidney tissues of LN patients and mice increased the expression of mammalian target of rapamycin (mTOR), resulting in exacerbation of intrarenal inflammation [80]. This was associated with activation of the TGF-β1/Smad/TLR3 pathway and renal fibrosis [81]. Alternative pathways may also contribute to renal fibrosis: miR-152 was downregulated in LN tissues and was negatively correlated with expression of MIF. The increased MIF expression in proximal tubular cells was associated with serum creatinine and 24-h urine protein, and also correlated with increased COL1A1 mRNA expression, a signature suggestive of EMT of injured renal tubular cells, resulting in extracellular matrix deposition and interstitial fibrosis. Interestingly, TGF-β1 levels were not affected, suggesting that MIF-induced EMT and fibrosis is a separate pathogenic pathway capable of causing chronic renal damage [86]. 

**Figure 2:**
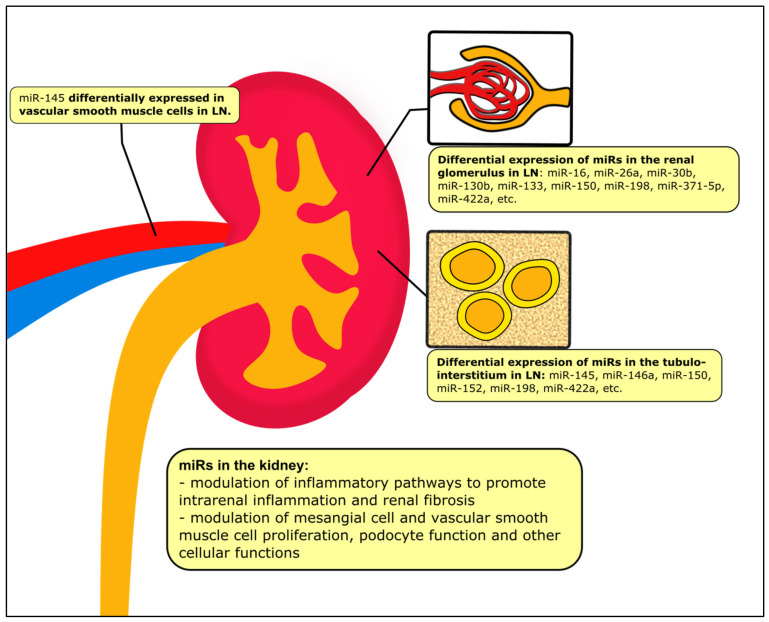
The role of miRs in different compartments of the kidney tissue in LN.

Compared to other analytes, miRs are especially useful in analysis of tissue samples: whereas mRNA is easily fragmented after formalin fixation, paraffin embedding and frozen storage, miRs are more durable and intact even in formalin-fixed paraffin-embedded (FFPE) tissue, possibly because of their shorter lengths [91,92]. In kidney tissues, ISH is conventionally used to localize the spatiotemporal expression of miRs [15], but novel methods such as laser-capture microdissection or fluorescent-activated cell sorting (FACS) may be capable of producing pure cell populations amenable to qPCR analysis [93]. Cell-specific miR expression is an especially important consideration in the kidney, as the profile of miR expression in the kidney in LN reflects not just immune dysregulation—the sine qua non of SLE—but also maladaptive responses specific to the kidney and its many different cell types, resulting in chronic inflammation and fibrosis, as illustrated in Figure 2. A crucial limiting factor of many studies is a lack of differentiation between the different histological classes of LN; while these are all largely due to immune complex and complement deposition, the pattern of cellular injury is different and likely associated with a particular miR signature. There is also insufficient comparative data to conclude whether the miRs affecting renal fibrosis and progression of CKD in patients with LN are different from those observed in patients with other causes of CKD. Further studies are clearly needed in this regard.

## 4. MiRs in the Urine

MiRs are secreted into various biological fluids including the urine, providing an easy, non-invasive means of diagnosing kidney pathology. In this case, miRs in the urine can be intracellular, or cell-free and bound to RNA-binding proteins or packaged into microvesicles, such as urinary exosomes [94]. While miRs may be filtered from the systemic circulation, many urinary miRs are released directly from nephron cells into exosomes, which are thus enriched in kidney-specific miRs [95]. MiRs within exosomes are endocytosed by recipient cells or recognized by receptors and may function in paracrine crosstalk between renal tubular cells. Thus, urinary exosomes in particular could potentially be utilized as a liquid biopsy to obviate the need for invasive kidney biopsies. 

During active flare of LN, let-7a and miR-21, miRs known to be differentially expressed in LN tissues and/or serum, were found to be downregulated in urinary exosomes [96]. Meanwhile, urinary exosomal miR-146a level was inversely correlated with circulating complement C3 and C4 levels, proteinuria and also CI in the kidney biopsy. Higher basal levels of miR-146a in urinary exosomes were associated with disease activity and could predict renal flares up to 36 months later. In vitro studies suggest that miR-146a in urinary exosomes may reflect a protective mechanism against NF-κB- and IFN-mediated podocyte inflammation and injury. Podocytes treated with LPS induced a rapid increase in miR-146a levels in the exosomal fraction, and intracellular mRNA of TLR4, TRAF6 and IRAK1 rose in tandem [97]. In a study of the urinary sediment of SLE patients, miR-146a was elevated, positively correlated with GFR, and inversely correlated with urinary expression of TNF-α, highlighting its role in regulation of glomerular inflammation. Likewise, miR-155 in the urinary sediment was correlated with the degree of proteinuria and SLEDAI scores [50]. MiR-221 and miR-222 in the urinary sediment were negatively correlated with anti-dsDNA levels in LN patients whereas urinary sediment miR-221 correlated with complement C3 levels, suggesting a role in the immunopathogenesis of LN [98].

Urinary miRs may also suggest particular histological features on the kidney biopsy. For example, in a small study of LN patients, total urinary miR-3201 and miR-1273e were reduced and associated with the presence of moderate-to-severe endocapillary proliferation, and could distinguish proliferative LN from non-proliferative LN; urinary miR-30c-5p was associated with proteinuria and cellular crescents [99]. A separate study showed that elevated miR-654-5p and miR-3135b in urinary exosomes distinguished class IV LN patients with cellular crescents from those without [100]. However, the exact roles these miRs play in glomerular inflammation are not clear, as their role in LN pathogenesis have not been previously described. As miR-654-5p was previously associated with proliferation of cancer cells in oral squamous cell carcinoma and prostate cancer; translated to the context of LN, it may regulate the abnormal proliferation of epithelial cells, a characteristic of cellular crescents in the Bowman’s capsule [100].

Urinary miRs have been associated with various kidney disease and renal fibrosis. In the context of LN, urinary exosomal miR-29c was negatively correlated with histological CI and with glomerular sclerosis. MiR-29c was not identified in the urinary cell pellet. Downstream transducers of the TGF-β1 pathway, including Smad3 and matrix metalloproteinase 2 (MMP2), were negatively correlated with miR-29c in urinary exosomes, while no correlation with estimated GFR or creatinine was observed [101]. Recognizing that serum creatinine, as well as other creatinine-based estimates of renal function including estimated GFR, are insensitive for early kidney damage, miR-29c in urinary exosomes may serve as a useful predictor of early renal fibrosis in LN. Taking this further, a combinatory panel of several urinary exosome-derived miRs was proposed to diagnose early renal fibrosis and predict progression to ESKD. As an individual biomarker, exosomal miR-150 was shown to have the best sensitivity and specific to distinguish between groups with high and low CI; this corroborates the previously described role of miR-150 in mediating renal fibrosis in kidney tissue from LN patients. Specificity for diagnosing early renal fibrosis further increased when urinary exosomal miR-29c and miR-21, another miRs involved in regulation of the TGF-β1/Smad3 pathway and implicated in other kidney diseases, were added to miR-150 [102]. 

Urinary miRs are clearly promising biomarkers for non-invasive diagnosis and prognostication. Unfortunately, many studies do not necessarily distinguish between miRs detected in the urinary exosomes, the urinary cellular pellet or the supernatant. This distinction is particularly pertinent to SLE: in patients with SLE, nearly all urinary miRs tested were found in exosomes, with significantly lower levels found outside them, especially in patients with active LN [103]. Similar to as with serum biomarkers, it is unlikely that a single urinary miR will be of sufficient diagnostic value to be of clinical use. Multi-marker panels will have to be designed and validated in larger studies for clinical application. In addition, in patients with more advanced stages of CKD, or who have concomitant kidney pathologies such as diabetic kidney disease, comparative studies are required to distinguish the miR signatures specific to active LN requiring targeted immunomodulatory treatment.

## 5. MiRs and the Treatment of LN

It is clear that miRs are critical to the pathogenesis of LN, and represent attractive targets for disease diagnosis, prognostication and treatment. Multi-marker panels using serum and urinary miRs can be used to diagnose, stage and prognosticate in LN. The association between miRs and specific signal transduction pathways means that immunosuppressive treatments can be tailored to a particular miR profile. For instance, the use of mTOR inhibitors is an emerging treatment for LN [104,105], and may be warranted in LN patients with downregulated miR-183, which was associated with over-activation of mTOR. Moreover, based on the associations described in previous sections, enhanced expression of miR-223 may suggest activation of S1pr1, for which a modulator, fingolimod, is currently commercially available; and clinical trials of a more specific modulator, cenerimod, is also underway in SLE patients (ClinicalTrials.gov identifier: NCT03742037). Furthermore, several studies have attempted to use direct administration of miR agomirs or antagomirs in murine LN models, with varying degrees of success. As miRs can affect the function of many cell types and disease processes including metabolism and carcinogenesis, the manipulation of miRs in humans should be exercised with caution to avoid off-target effects. Exogenously administered miRs are also not immediately bioequivalent to endogenously produced miRs. As a tale of caution, experimental transfection of miR mimics into human cells led to non-specific changes in gene expression and the accumulation of high-molecular weight RNA species [106]. Clinical trials should thus address both the short- and long-term safety of modulating miRs in LN patients. Targeted delivery systems for the treatment of organ-specific disease, such as in the case of LN, may also help avoid untoward systemic effects of miR treatments. Since lupus susceptibility genes may be targeted by multiple miRs, it is also worthwhile to investigate the efficacy and tolerability of combination miR treatments.

In addition, much research will be needed to determine the effect of immunosuppressive treatments on miR expression in various body compartments. As an example, mycophenolic acid (MPA) was shown to upregulate the depressed miR-142-3p/5p and miR-146a levels in CD4^+^ T cells in SLE, though the miR expressions were not restored to that in healthy donors, suggesting presence of other factors not targeted by MPA [107]. Other drugs commonly used to treat SLE, including dexamethasone, cyclosporine A and methotrexate, did not affect the expression levels of these miRs. In another study, miR-146a was also not affected by treatment by prednisone, hydroxychloroquine, or mycophenolate mofetil or combinations of these treatments [108]. Other investigators showed that treatment with rituximab in refractory SLE was able to reverse the differential serum miR signature observed in active SLE, validating a 5-miR panel consisting miR-149-3p, miR-125b-5p, miR-199a-5p, miR-106b-3p and miR-124-3p [109]. In a murine model of SLE, treatment with hydroxychloroquine increased the expression of miR-590, an miR involved in T_H_17 differentiation, in T_H_17 cells, and this was correlated with reduced IL-17 secretion [110]. Urinary miR-let-7a, miR-21 and miR-155 were downregulated following treatment with prednisone and hydroxychloroquine in mice [111], and another study showed urinary miR-155 was reduced by calcitriol treatment, although these results have not been reproduced in other studies [50]. These changes in miRs expression following the use of immunomodulatory therapies may provide new insights on the mechanisms of action of these medications, and may serve as a novel functional biomarker to monitor the immunomodulatory efficacy of these medications. Further studies correlating miRs and other epigenetic factors with the effects of immunomodulatory treatments will be of paramount importance, in order for the clinician to understand how to use miRs at different stages of LN, especially in patients who have received intensive treatments previously or are refractory to standard therapies.

## 6. Conclusions

The role of miRs as key epigenetic regulators in LN is increasingly recognized. The available evidence suggests that miRs are involved in LN pathogenesis not just by modulation of immune cells and systemic inflammatory pathways, but also by acting at the level of the kidney tissue and even in the urinary exosome. Further translational research could transform miRs into useful diagnostic and therapeutic tools for patients with LN and address major unmet needs in this patient population. 

## Figures and Tables

**Table 1:** Signal transduction pathways involved in the pathogenesis of LN and the expression of relevant miRs in the circulation and PBMCs.

Associated Pathways	Relevant miRs in the Circulation and PBMCs	Change in miR Expression
NF-κB/IFN-1	miR-146a [20,21,22,24,26,27,50,51] miR-124 [28] miR-199a-1 [19] miR-125b-5p [52]	conflicting decrease increase increase
MIF	miR-654 [29] miR-451a [30]	decrease decrease
PTEN/FoxO Smad/TGF-β	miR-21 [31,32,33] miR-182-5p	increase increase
PDCD4/IL-10	miR-21 [31,32,33]	increase
T cell function/differentiation	miR-21 [33] miR-148a [33] miR-10a-3p [34] miR-224 [35] miR-371b-5p [36] miR-5100 [36] miR-223 [37] miR-155 [17,23,27,31,38]	increase increase decrease increase increase increase decrease decrease
B cell	miR-155 [17,23,27,31,38] miR-29c [39] miR-145 [39,40] miR-15b [38,41] miR-148a [43] miR-22 [38,39] miR-18b [38,39] miR-345 [38,39] miR-365 [38,39]	decrease increase increase decrease increase increase increase increase increase

FoxO, forkhead box O; IFN-1, Type 1 interferon; IL-10, interleukin-10; MIF, macrophage migration inhibitory factor; NF-κB, nuclear factor kappa-light-chain-enhancer of activated B cells; PDCD4, pro-inflammatory protein programmed cell death 4 (PDCD4); PTEN, phosphatase and tensin homolog; TGF-β, transforming growth factor-β.

**Table 2:** Signal transduction pathways involved in the pathogenesis of LN and the expression of relevant miRs in the kidney tissue.

Associated Pathways	Relevant miRs in the Kidney Tissue	Change in miR Expression
NF-κB	miR-199a [69] miR-let-7a [70,71] miR-let-7e [70] miR-663a/miR-423-5p [72]	increase increase increase increase
IFN-1	miR-130b [73,74] miR-26a [75] miR-30b [75]	conflicting decrease decrease
PTEN	miR-130b [73,74] miR-198 [51,76]	conflicting increase
TGF-β1	miR-150 [77,78] miR-410 [79] miR-183 [80,81]	increase decrease decrease
Other cellular proliferative and inflammatory pathways	miR-133 [82] miR-16 [83] miR-371-5p [84] miR-146a [51] miR-422a [59] miR-145 [85]	decrease decrease decrease increase increase decrease
Other fibrotic pathways	miR-152 [86]	decrease

IFN-1, Type 1 interferon; NF-κB, nuclear factor kappa-light-chain-enhancer of activated B cells; PTEN, phosphatase and tensin homolog; TGF-β1, transforming growth factor-β1.
